# Libel Actions by Medical Practitioners

**Published:** 1898-02-19

**Authors:** 


Vol. XXIII.—No. 595]. Edited by Sir Henry Burdett, K.C.B., and Solomon C. Smith, M.D., M.R.C.P. [Feb. 19, 1898.

### Libel Actions by Medical Practitioners.

It has often been declared by eminent members of the legal profession that they would rather submit to almost any injury or indignity than seek the compensation likely to be afforded to them in a court of law; and a case which was heard at the Manchester Assizes on Friday, before Mr. Justice Grantham and a special jury, certainly affords some ground for the opinion that the prevailing practice of lawyers with regard to litigation in which they are personally concerned might be followed by the members of other professions with every prospect of advantage. According to the *Times* report, Dr. Kingsbury, of Blackpool, sued a Mr. Willoughby, a Manchester architect, for slander. It appeared that in August last the defendant was staying at the Imperial Hotel in Blackpool, and that he became acquainted with a lady named Pollock, who was staying there. Mrs. Pollock and Mr. Willoughby were in conversation in the hall of the hotel when the plaintiff passed through it, and it was alleged that they began to talk about him, and that the defendant made statements to his disadvantage. It is not shown by the report that any special damage was either suffered or claimed for; and, indeed, it is stated not only that Mrs. Pollock was the plaintiff's patient, and presumably continued to be so, but also that she was his informant with regard to the injurious words which had been spoken of him. The defence was that the words really used had been exaggerated in repetition, that Mrs. Pollock asked the defendant's opinion of the plaintiff, and that the defendant conceived the occasion to be a privileged one, on which he was entitled to say whatever was in his mind. The judge, it need hardly be said, at once disposed of the dangerous plea of privilege, as created by a casual question; and the jury, after a brief absence, found for the plaintiff, with a pound damages. No decision was announced as to costs, which, in such a case, would be wholly at the discretion of the judge; but, even if they should be given to the plaintiff, they will not be in the least likely to cover his expenses.

There is a venerable inscription, "They say! What say they? Let them say!" carved, if we remember rightly, over the principal gateway of Marischal College, Aberdeen, which every medical practitioner would be wise to take as his motto, and to adopt as a principle governing his conduct. It is fortunately rare for members of the sterner sex to let their tongues run away with them concerning professional practitioners of any kind, legal, medical, or other; and it is highly probable that Mr. Willoughby has already deeply repented of his indiscretion. But only think of "pretty Fanny's way" with her doctors in general, and especially of the way of those Fannies who live in the suburbs of great towns in which their husbands are engaged, and who come together over five o'clock tea. What subject of discourse is half so fascinating, next to their actual maladies, as the manners and customs and opinions of the several competing practitioners of the neighbourhood. It is a common habit with such ladies, we have been credibly informed, not only to extol their own doctors to the skies, but to describe all others as imbeciles; an arrangement under which each follower of Æsculapius may reasonably hope to be supported by at least one champion in every party at which he is discussed. The general effect is, we believe, precisely *nil*. Every speaker knows in her heart how slender is the foundation for her own fault finding, and allows a liberal discount for the fault finding of others. The praise, it is easy to believe, generally rests upon surer ground, inasmuch as there are very few people who have not experienced kindnesses from their doctors on which they like to dwell, and which will predispose them to give credence to the favourable reports of others. How much better it would have been if Mrs. Pollock had been content with defending her friend on the basis of her own experience of his skill, and had refrained from carrying idle tittle-tattle to his ears. Would it not also have been better if Dr. Kingsbury had been content to leave the injurious statements to be rebutted by his own character, from which they would, no doubt, have fallen quite harmlessly. We do not believe that any man can be seriously injured in his professional practice by anybody but himself; and we do not think it is judicious to be too sensitive to the casual prattle of people who, in relation to medical skill, must necessarily be ignorant, and who, in relation to charges, often take a much less liberal view of the doctor's claims than they would do of any similar question affecting themselves. Sir Thomas Watson long ago pointed out that the doctor, like Belinda's Betty, is often "praised for labours not his own;" and it is, perhaps, the part of sound judgment to allow both undeserved praise and unjust censure to pass like the wind, which bloweth where it listeth, and of which no man can tell whence it cometh or whither it goeth.

